# A Custom-made Pupillometer System for Characterizing Pupillary Light Response

**DOI:** 10.4274/tjo.39049

**Published:** 2018-09-04

**Authors:** Nefati Kıylıoğlu, Mahmut Alp Kılıç, Tolga Kocatürk, Seyhan Bahar Özkan, Mehmet Bilgen

**Affiliations:** 1 Adnan Menderes University Faculty of Medicine, Department of Neurology, Division of Clinical Neurophysiology, Aydın, Turkey; 2 Adnan Menderes University Faculty of Medicine, Department of Ophthalmology, Aydın, Turkey; 3 Adnan Menderes University Faculty of Medicine, Department of Biophysics, Aydın, Turkey

**Keywords:** Pupillary light reflex, pupillary response, pupillometer

## Abstract

**Objectives:**

This paper presents the design and construction of a viable pupillometer system and demonstrates its merits with extensive validation tests.

**Materials and Methods:**

A web camera was modified by removing its infrared filter and mounted on a chin rest. Light emitting diodes (LEDs) operating at infrared and visible spectra were integrated to provide background and light stimulus, respectively. The LEDs were controlled by a microprocessor board. Stimulation was presented using a periodic paradigm with variable period and duty cycle. Videos of both pupils were recorded at 30 frames/second and processed offline using software developed in-house. The overall system was validated with data gathered from individuals with healthy vision under different stimulation paradigms. Temporal variations in pupil size were determined and analyzed statistically.

**Results:**

The analysis revealed that the pupil sizes were accurately measured from the video frames provided that reflections from both infrared and visible lights remain outside the pupil. The system achieved moderate to excellent repeatability scores (87.8 and 86.8% for short 1 second and long 2 second pulses, respectively), which demonstrated its effectiveness and confirmed that it can be used reliably as a pupillometer.

**Conclusion:**

The proposed pupillometer system produces useful, quantitative data characterizing pupillary light response. However, further development and implementation are needed to potentially turn it into a low-cost alternative for other studies involving the autonomic nervous system, cognitive function, drug metabolism, pain response, psychology, fatigue, and sleep disorders.

## Introduction

The pupil is an important functional structure that balances the amount of illumination entering the eye to enable clear vision. The sphincter pupilla and dilator pupilla muscles act together under the control of the parasympathetic and sympathetic nervous systems. The muscles are respectively governed by the oculomotor nerve, sympathetic nervous system tracts and fibers that are located in the mesencephalon and the cervical spinal cord. The functional status of these structures (optic nerve, mesencephalon, spinal cord, oculomotor nerve, cervical sympathetic fibers, and pupillary muscles) is evaluated by the pupillary light response (PLR).^1^ Although physical examination of PLR is the usual method of evaluation, use of a device called a pupillometer offers more diagnostic sensitivity than physical examination alone.^2^ Commercial pupillometers are both costly and unavailable in clinical practice, especially in developing countries. Also, they lack flexibility and versatility for research purposes where light stimulation under different paradigms may be required. This paper addresses this issue. We have developed a viable pupillometer system for real-time video recording of pupil response to light stimulation and video analysis software for characterizing PLR. The overall system was validated and its merits were investigated with the data acquired from healthy individuals subjected to periodic white light stimulations with short and long pulse durations.

## Materials and Methods

### Video Acquisition and Recording Hardware

**Figure 1:**
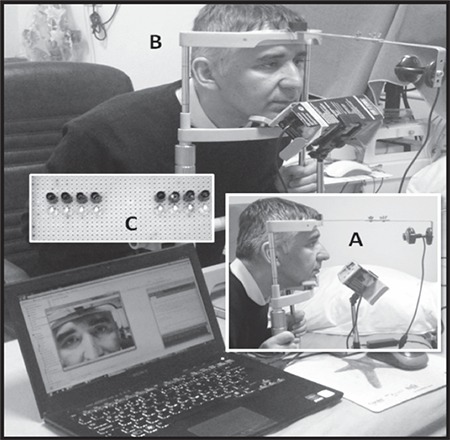
Custom-made pupillometer system used in this study. A) Lateral view, B) Antero-lateral view, C) Placement of infrared (lower) and white light diodes (upper) from the front view

Video acquisition and recording functions in the pupillometer system were achieved in a cost-effective manner using a common web camera (INCA_IC-3562 model) attached to a standard laptop PC via a USB cable and a software package (AMCAP Version 8.11). The camera was modified by removing the infrared filter in front of its lens. This expanded the operation of the camera into a near-infrared spectrum and allowed visualization of the pupil in both dark and light conditions. The frame rate of the camera was set at 30 frame/second, but the actual rate was determined to be 25 frame/second for the real video recordings in mpeg4 format. During the examination, the subject sat on a chair in an upright position, placed his/her chin on a metal structure to which the camera was mounted, and was asked to focus on the camera (Figure 1). Eye-to-camera position was carefully leveled to remain horizontal, and the distance between was maintained at 24 cm.

### Infrared and White Light Setup

The stimulation arrangement consisted of infrared and white light emitting diodes, placed separately on a specially designed circuit board. Four diodes were placed side-by-side as a bank. White lights were positioned below the infrared diodes. The printed circuit board spanned both eyes and was placed near the cheek, below the eyes. It was oriented at an angle of approximately 130-140° to the horizontal, sloping away from the subject. This orientation prevented white light reflections as they remained below the pupil during the recordings. Otherwise, the reflections within the pupil interfered with the process of pupil size estimation during the off-line video analysis. This approach improved the accuracy of the estimates calculated using semi-automatic software developed in-house as discussed below.

Both the white light and infrared diodes were connected electrically to a microcontroller board (Arduino UNO). It was programmed (Arduino 1.6.0) to carry out the specific stimulation paradigms by periodically turning the white light diodes on and off for a predefined duration while leaving the infrared diodes on for the entire recording time. The peak wavelength of the infrared diode was 940 nm. Turning this diode on alone did not induce any pupillary reaction nor interfere with the eye response induced by the white light.

In a PLR examination, both eyes were stimulated simultaneously with light-dark periods and the responses of both pupils were recorded throughout the session. The resulting video was appropriately named and stored digitally for post-processing.

### Video Processing Software

**Figure 2:**
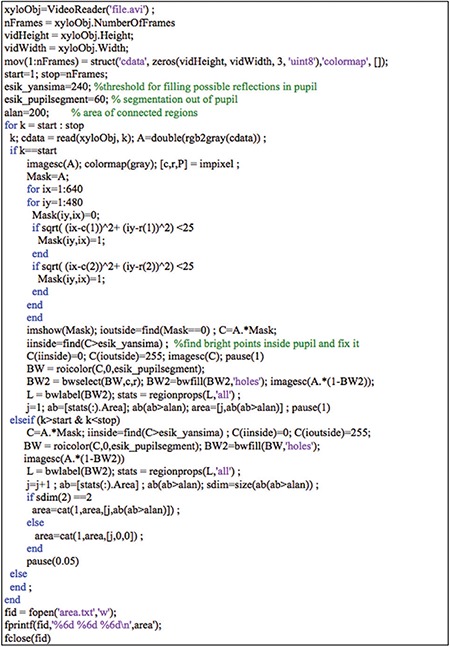
Matlab code for video processing to estimate pupil sizes in both eyes. The variables "esik_yansima", "esik_pupilsegment" and ‘alan’ in the program may require adjustments based on the purpose of the video analysis in order to improve the accuracy of the pupil size estimates

The video files were analyzed using software developed in the Matlab environment (Matlab Version R2015A, The MathWorks Inc., Natick, MA). The Matlab code is given in Figure 2. The program runs in semiautomatic mode. The code opens the video file, starts with a predefined video frame, and lets the user manually mark the centers of the pupils in both eyes. The pupils in the first and the following frames were identified automatically. Also, the pupils were segmented out and their sizes were calculated pixel-wise, both automatically, in each video frame and recorded sequentially in a text file along with the frame number. Estimating pupil size was not possible in some frames due to eye blinking or closing. These data points were filled with zero automatically.

### Validation Measurements of Repeatability

The light stimulation paradigms used for validation purposes involved periodic short 1 second light/1 second dark or long 2 second light/2 second dark pulses. Each recording started with a baseline acquisition of 5 second followed by at least 12 periods of light/dark stimulus cycles. Under each paradigm, the intensity produced by white light was measured using a power meter (Lutron-Model LX-1108) which was placed at the same level as the left eye. During the examination, videos were acquired with both 1 second and 2 second stimulation paradigms. To test repeatability, the same paradigm was repeated in each session with a 2 minute rest interval in between. The videos were processed off-line using the code in Figure 2. The pupil size estimates for both eyes in the frames under each stimulation paradigm were sequentially stored in text files.

### Ethical Issues, Inclusion and Exclusion Criteria

The study was approved by the Medical Ethics Committee of Adnan Menderes University (2015/577). Signed consent forms were obtained from the volunteers. Inclusion criteria were: having no history of any previous disorders which caused transient visual loss, being free from any medication, and having no sleep complaints. After being informed about the procedures, volunteers underwent a full ophthalmological evaluation either between 10:00 and 12:00 am or between 1:00 and 4:00 pm.^3^ Those with a best corrected visual acuity of 20/20 in both eyes were subjected to further examination for PLR. All volunteers completed the evaluation process.

### Temporal Analysis of Pupil Size Changes with Light Stimulus

The text files were analyzed in Excel (MS Office) and the temporal data were plotted as a function of frame number. Data series with zero values or those identified with eye movement or with abnormally high or low values were excluded from the analysis. Once satisfied with the behavior of the displayed graphs, we went back and normalized the pupil size measurements frame-by-frame by the baseline recording (averaged over 5 s of time duration). That is, all pupil size estimates including the baselines were scaled according to the following formula:^4^

Normalized pupil area (NPA) = (Average baseline - Pupil size)/Average baseline x 100.

The resulting NPA readings were equivalent to the percentage reduction in pupil size. Normalization also reduced the effect of aging on the temporal dynamics of pupil size, according to a previous report.^5^

Next, the time signals were consecutively extracted period by period (i.e. over the total duration of 12 consecutive light and darkness) and the segments were further averaged temporally to obtain a single trace of pupil response profile for each eye. This approach eliminated the variation in pupil response with the stimulus cycle. The resulting signal profile was described by a set of parameters which were then measured from the data gathered from all research participants and further analyzed statistically.

### Statistical Analysis

The data included temporal variations of NPA. The periodic signals obtained from both right and left eyes under the stimulation protocols were investigated for compatibility, consistency, and repeatability by intra-class correlation coefficient test (single-measurement, absolute-agreement, 2-way mixed effects model).^6^

## Results

**Figure 3:**
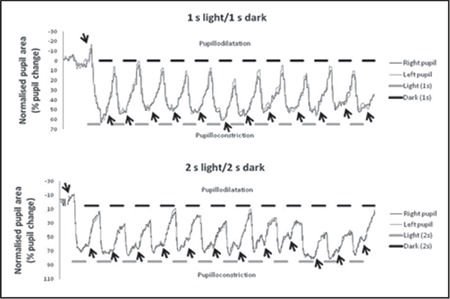
Temporal profiles of normalized pupil areas in both eyes under the stimulation paradigms as encoded by the black and gray bars. The single arrow at upper left points to small pupil dilatation when the lights were off and the lower twelve arrows point to pupil constrictions when the lights were on

Thirty-seven volunteers, 16 females (age: mean 34; min 20 and max 61 years old) and 21 males (age: mean 36; min 20 and max 60 years old), were included in this study. The test runs with the stimulation paradigms producing light intensities listed in Table 1. The intensity values represent the average over 12 periods of dark and light durations. The light levels achieved with the paradigms were sufficiently high to induce strong pupil responses. Figure 3 shows the representative graphs of NPAs as obtained from an examination session with the procedures described above. In both eyes, NPAs exhibited identical behaviors, indicating the capability of the system to promptly follow changes in pupil size in response to the light stimulus.

Intra-class correlation coefficient calculations from the first and second trials under both paradigms are summarized in Table 2. Most cases had moderate to excellent repeatability scores (87.8% for the 1 second and 86.8% for the 2 second stimulation paradigms). These findings confirmed the quality of the match between the signal pairs obtained with repetitions.^6,7^

**Figure 4:**
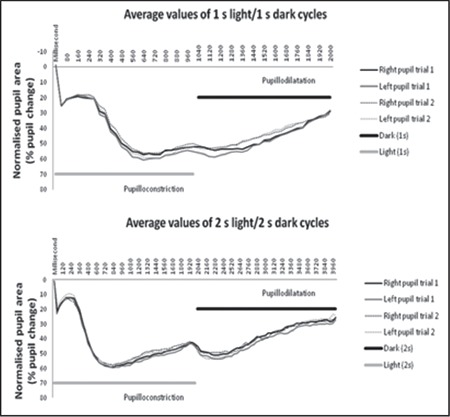
Mean response profiles of pupil size to 1 second light/1 s dark (top trace) and 2 second light/2 second dark (bottom trace) stimulus durations as encoded by the black and gray bars

Figure 4 shows representative mean traces (average over 12 cycles) of the pupil response in Figure 3. The corresponding plots for each eye also exhibit very close traces.

## Discussion

In spite of commercial availability, there are still attempts to build custom-made pupillometers to address specific concerns.^7^ Parameters such as initial pupil size and duration/velocity/latency of pupillary contraction and dilatation are of clinical interest as they reflect the functional state of the eye. Classically, the V-shaped response was observed with the light stimulus. When the light was on, the pupil first contracts and then dilates after the light turns off (Figures 3, 4). The temporal appearances of PLR traces for the 1 second and 2 second stimulus paradigms were similar to those produced by the commercial pupillometers.^7^ However, while commercial devices typically produce data from a single stimulus with a fixed period and duty cycle, our system is capable of handling periodic stimulations with different periods and duty cycles. The system is also flexible in the sense that white diodes can be replaced by those with different colors of interest to facilitate PLR studies concerning color dependence. Moreover, the continuous stimulation does not lead to habituation.

Eye movements (saccades) affect video-based estimates of pupil size, especially when a computer screen is used for stimulation purposes.^8^ In our setup, the patient was asked to focus on the camera’s shutter during the examination. The software monitored the stability of the eye by tracking the center of mass of the pupil segment in each frame. Positional shifts of more than 10 pixels in length were considered as indicative of eye movement. These efforts ensured increased accuracy in the pupil capturing area. However, in a very small number of cases, data gathering and analysis were limited due to low eyelid position, frequent blinking, insufficient data capturing of pupil area because of interference with shadow due to light-dark cycle, and also interference related to make-up in female subjects. This is why the total numbers in the frequency column of Table 2 do not add up to 74 (37 participants with 2 eyes), meaning that it was not feasible to estimate the pupil size even after filling the missing data points with zero. Nevertheless, in the absence of such issues, the custom-made pupillometer and video analysis software platform developed in this study revealed moderate to excellent repeatability scores (greater than 85% of the people investigated) and its performance was comparable to the test-retest repeatability of the previous pupillometer scores.^2,7^ Lei et al.^7^ evaluated the test-retest reliability of hemifield, central-field, and full-field chromatic pupillometry. For the post-illumination pupil response, they determined intra-class correlation coefficients of 0.84 (0.69-0.95) and 0.94 (0.83-0.98) at full field stimulation with blue light. Unlike our study, which did not assess interobserver variability, Couret et al.^2^ investigated the interobserver variability and reported intra-class correlation coefficients of 0.95 and 0.87 for pupil size at both resting and after light stimulation, respectively. Our intra-class correlation coefficient values were similar, confirming that the pupillometer system can be used reliably to evaluate PLR.^6^

The potential value of PLR evaluations in normal and disease conditions have been investigated in many studies in areas such as the autonomic nervous system, cognitive function, drug metabolism, pain response, psychology, fatigue, and sleep disorders.^9,10,11,12,13,14,15,16,17,18,19,20^ There is a growing interest in diagnosis in these areas, and our custom-made pupillometer may be useful as an easily-applicable, non-invasive diagnostic tool.

### Study Limitations

As mentioned earlier, in a few cases there were limitations in data gathering and analysis due to low eyelid position, frequent blinking, insufficient data capturing due to shadowing, and interference related to cosmetics worn by female subjects. Extracting data from the videos was also time-consuming.

## Conclusion

Our study demonstrates that our custom-made pupillometer and video analysis software platform can be used to reliably evaluate PLR. However, further development and implementation are needed to potentially turn it into a low-cost alternative.

## Figures and Tables

**Table 1:**
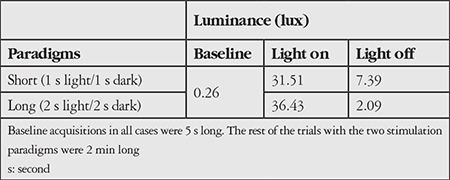
Light intensities measured under each light stimulation paradigm

**Table 2:**
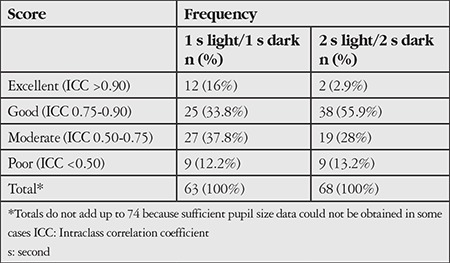
Intraclass correlation coefficient values calculated from the normalized pupil area signal pairs (first and second trials) gathered from all subjects (n=37) for both eyes (74 eyes)
